# Asexualization for the Prevention of Crime and the Curtailment of the Propagation of Criminals

**Published:** 1894-09

**Authors:** F. L. Sim

**Affiliations:** Memphis, Tenn.


### ASEXUALIZATION FOR THE PREVENTION OF CRIME AND THE CURTAILMENT OF THE PROPAGATION OF CRIMINALS.*

BY F. L. SIM, M.D.,
MEMPHIS, TENN.

Capital punishment is a curse to our nation, as it has been to every government on earth that has practiced it. Judical homicide and legalized murder are names that place the hanging of criminals in line of proper nomenclature. Reasons for such conclusions are obvious. The following are some of them:

1. A number of instances can be cited to prove that the abolition of capital punishment has not been followed by the increase of crime. Rhode Island abolished capital punishment fifty years ago; Michigan and Wisconsin twenty years ago. It has been practically abolished in Kansas for fifteen or twenty years, not by statute, but by the custom of governors in failing to fix the date of execution, and in neither of these States do the figures indicate an increase of crime as the result of such abolition.

2. It is the *certainty* of punishment, rather than its severity, that deters men from committing crime. Nearly all criminals hope to escape, and most of them do escape through legal technicalities and the growing disinclination to enforce the extreme penalty. Hence capital punishment is even now practically abolished, a dead letter on the statute books. We compile the following figures from the report of the United States Census Office for 1890: Of 7,386 prisoners charged with homicide, 158 were executed, or only 2.13 per cent. Where statutes exist providing capital punishment for murder, they are not only to a great extent inoperative, but they absolutely shield a large per cent. of the most desperate criminals from any and all punishment.

---

*Abstract of paper read before Tennessee Medical Society, April, 1894.

3. Executions partake of a spirit of revenge often, if not always, and cultivate a feeling in the community akin to that possessed by the criminal when committing crime.

4. The taking of life is often more than the demands of society render necessary, provided other punishment could be substituted.

5. It is certain that other punishments, equally applicable and far more scientific, can be substituted, and at the same time place a lasting and effective object-lesson before the community.

6. Aside from the demoralizing influences of executions, it is well known that numerous innocent lives have been sacrificed through the fallibility of evidence.

The most potent factor that now sustains capital punishment is, probably, the belief that it is the severest that can be inflicted, as this so-called punishment rids the man of his life and the community of the man. The criminal, if prepared to go, a regeneration which most of them claim, is only cheated of a part of his miserable life and is hurried to "eternal glory." Even the *methods* of execution are now becoming repugnant to the people, and it is sought to glide the criminal, on "flowery beds of ease" over the Jordan, with the aid of electricity or anesthesia.

The desideratum of the hour is a substitute for capital punishment, now almost a farce with the American people.

Tennessee makes rape punishable with death, but this is too good for such a class of men, and the tortures of former times too demoralizing upon the community for their rehabilitation. Castration and the penitentiary are their just dues.

Sterilize criminals, confine them to the penitentiary for a term of years, and no ill effects from a moral point of view will follow; while it fully protects from further crime originating in sexual disease, and shields society from their like forever. When the question of insanity is urged in justification of crime, and such mental condition is due to sexual excess, sexual sterilization, whether the criminal be male or female, should undoubtedly be a part of the remedial agency punishment prescribed.

Would castration be a grave punishment to men? Castration among the moral, for disease, is grave enough, but with him who devotes his entire thought to sexual gratification, death is secondary to sterility. Sexual debauchery is world-wide, both in its existence and in its crime and disease producing characters. The appetite is easily cultivated, and *pari passu*, with its growth are the powers of restraint weakened and the nervous system involved. Hence men frequent the gambling table, become defaulters, robbers and murders in securing the means of gratifying their sexual appetite and the cravings of torturing nervous systems for alcoholic anesthetization. Step by step the line of sexual debauchery is pursued until a grave crime follows and the subject confronts the law.

In the scientific handling of all criminals, whose crimes are associated with or dependent on sexual depravity, such persons would keenly feel the awful justice of the remedial agency, and would appreciate a life of rectitude should they regain their freedom. The history of the eunuch of former times shows that he not only lost his licentiousness, but all feeling of manhood and equality as well, and grew fat from an easy conscience and a tranquil nervous system.

Sexual sterilization undoubtedly robs the subject of all hope in this world, but it leaves intact his prospects for a life of virtue and happiness in the great beyond. It is to the prevention of such crime that we must bend our energies if we would save the community.

One has only to look over the current literature of the day, North and South, for assurance that the black people are rapidly coming to the front as criminals. The frequency of the crime of rape perpetrated upon the white female is becoming paralyzing to the country, and yet provisions of our law, making such attacks felony punishable with death, coupled with the terrors of the mob, have not only failed to check but have actually permitted an increase of the crime.

A prominent attorney of the law recently informed the writer that certainly not more than one-fifth of the rapes committed by negroes with their own color ever come to light. A free dispensary physician states that application for remedial agents to relieve the suffering following rape or attempt thereat are by no means uncommon.

If the law provided sexual sterilization for all such rascals, the colored man would soon know that rape is a crime of no small magnitude even with its own color. Then, too, it would place an object lesson, a priceless and demure eunuch, within every negro community in the Southern States.

In the opinion of the writer the method of scientific inquiry into the causes leading on to the committal of crime and the adaptation of remedial agents that render such cases subsequently inoperative, either with or without additional punishment, is the proper method for the immediate and permanent protection of society, the punishment of criminals and the arrest of their propagation.

---

### ACUTE TONSILLITIS.

Salicylate of soda is, according to the *Northwestern Lancet*, often very little less than a specific. Its use should begin as early in the attack as possible, and should be pushed to the point of producing slight ringing in the ears, when the dose may be decreased, but its administration may be kept up for a day or two after the disappearance of the fever. Fifteen grains every three hours will, in most cases, cause the ears to ring, when ten, and later five, grains may be given at the same interval.

The five grain compressed tablet is a convenient form of administration, and if the dose causes nausea a few swallows of milk taken with it will usually prevent this complication.

---

### CHAPPED HANDS AND FACE.

Dr. W. P. Spratling, in the *Medical Record*, says a most excellent remedy for chapped hands and face, and one that, if properly used, will cure the most painful cases in from twelve to twenty-four hours, is compounded as follows :

℞ Tr. benzoin comp.......................................*m* x.

Alcohol.......................................ʒ ij.

Aqua rosæ.......................................*m* xxx.

Glycerine.......................................q. s. ad. ℥ j

M. SIG.—Apply to chapped surfaces at night after they have been washed with soap and warm water and thoroughly dried.

This remedy is equally efficacious in the treatment of fissured, bleeding and sore lips.

